# Sensitization of lung cancer cells by altered dimerization of HSP27

**DOI:** 10.18632/oncotarget.22192

**Published:** 2017-10-31

**Authors:** Byeol Choi, Seul-Ki Choi, You Na Park, Soo-Yeon Kwak, Hwa Jeong Lee, Youngjoo Kwon, Younghwa Na, Yun-Sil Lee

**Affiliations:** ^1^ Graduate School of Pharmaceutical Sciences, Ewha Womans University, Seoul 120-720, Korea; ^2^ College of Pharmacy, CHA University, Pocheon 487-010, Korea

**Keywords:** HSP27 inhibition, altered dimerization, combination therapy, HSP27 inhibitor

## Abstract

Heat shock protein 27 (HSP27, HSPB1) induces resistance to anticancer drugs in various cancer types, including non-small cell lung cancer (NSCLC). Therefore, pharmacological inhibition of HSP27 in NSCLC may be a good strategy for anticancer therapy. Unlike other HSPs such as HSP90 and HSP70, small molecule approaches for neutralization of HSP27 are not well established because of the absence of an ATP binding domain. Previously, small molecules with altered cross linking activity of HSP27, were identified to inhibit building a large oligomer led to sensitization in combination with radiation and chemotherapeutic drugs. In this study, a chromene compound, J2 that exhibited better cross-linking activity of HSP27 than xanthone compound, SW15 which was previously identified, was yielding sensitization to NSCLC cells with high expression of HSP27 when combined with HSP90 inhibitor and standard anticancer modalities such as taxol and cisplatin. *In vivo* xenograft system also showed sensitization activity of J2, as well as *in vitro* cell viability, cell death or apoptosis detection assay. For better druggability, several quinolone compounds, an (bio) isostere of chromone and one of well-known core in many marketed medicine, was designed and synthesized by replacement of oxygen with nitrogen in 4-pyron structure of J2. However, the cross linking activity of HSP27 disappeared by quinolone compounds and the sensitizing effects on the anticancer drugs disappeared as well, suggesting oxygene moiety of 4-pyron structure of J2 may be a pharmacophore for induction of cross linking of HSP27 and sensitization to cancer cells. In conclusion, combination of chemotherapy with small molecules that induces altered cross-linking of HSP27 may be a good strategy to overcome the resistance of anticancer drugs in HSP27-over-expressing cancer cells.

## INTRODUCTION

Heat shock protein 27 (HSPB1, HSP27 in human and HSP25 in murine) is a member of small HSPs and ATP-independent chaperons. HSP27 is presented in all human tissues, including primary neuronal cells and astrocytes but mainly in cardiac, skeletal muscles [1]. Its structure contains an NH2-terminal sequence including WDPF-motif, conservative α-crystallin domain, and a short COOH-terminal sequence. Both WDPF-motif and α-crystallin domain seem to be crucial for oligomerization. The α-crystallin domain contains single Cys residue (Cys137) that plays an important role forming disulfide bonds leading to accumulation of cross-linked HSP27 dimers. HSP27 is highly homologous with other small HSP27 family, containing the conserved α-crystallin domain and differing in the C- and N-terminal regions [2–5].

Over-expression of HSP27 suppresses apoptotic cell death caused by various stimuli, including oxidative stress, anticancer drugs. High levels of HSP27 are found in many cancer types, and clinical trials have revealed the relationship between HSP27 and aggressive cancers, metastasis, promoting drug resistance, and poor patient outcomes [5–11].

Lung cancer is the most universal cancer and the leading cause of cancer-related death worldwide. Treatments of lung cancer include surgery, chemotherapy, radiotherapy and targeted therapy. Types of lung cancer are classified in non-small cell lung cancer (NSCLC) and small cell lung cancer (SCLC). NSCLC accounts for approximately 85% of all lung cancers. Moreover, HSP27 are reported to be highly expressed in human NSCLC [7], suggesting that inhibition of HSP27 expression in NSCLC may be a good strategy for cancer therapy [8].

Because HSP27 is over-expressed in most cancers, it is an attractive target for cancer therapy. However, unlike HSP90 or HSP70, it lacks an active site or ATP-binding pocket. Hence, only two HSP27 inhibitors are in the clinical trials. One is antisense oligonucleotide Apatorsen (OGX-427), which targets the translation initiation site of human *hsp27* and inhibits the translation of mRNA of HSP27, thereby preventing the protein expression levels compared to untreated cells [11]. Second one is RP101 (bromovinyldeoxyuridine, brivudine), which is a nucleoside that binds via π-stacking with Phe29 and Phe33 of HSP27 thereby inhibiting its function. Their functions are chemosensitizing and inhibiting the development of resistance [8]. However, the limitations such as intracellular delivery challenge in the case of OGX427, so not competitive to a small molecule and lack of exact mechanism that acts on HSP27 in the case of RP101, are suggested [8–11]. Aside from RP101, no small molecules have been developed as HSP27 inhibitors for cancer therapy, although functional HSP27 inhibition may be a good strategy for combination therapy with HSP90 inhibitors, or chemotherapeutic agents.

We previously demonstrated that zerumbone (ZER), a cytotoxic component isolated from a natural product, *Zingiber zerumbet*, and SW15 a synthetic xanthone compound, induced cross-linking of the HSP27 protein by insertion between the disulfide bonds of HSP27. Altered cross-linking of HSP27 modified normal HSP27 dimerization, which resulted in a sensitizing effect to tumors after treatment with radiation (IR) [12, 13]. Therefore, altered cross-linking strategy was suggested as a novel strategy for inhibition of HSP27-mediated resistance. In this study, we identified a more potent HSP27 cross linker, J2, a synthetic chromone compound, as well as its pharmacophore structure. Moreover, J2 also showed potent synergism with a HSP90 inhibitor or conventional anticancer drugs.

## RESULTS

### J2 with chromone structure showed strong altered cross linking activity of HSP27

**Figure 1:**
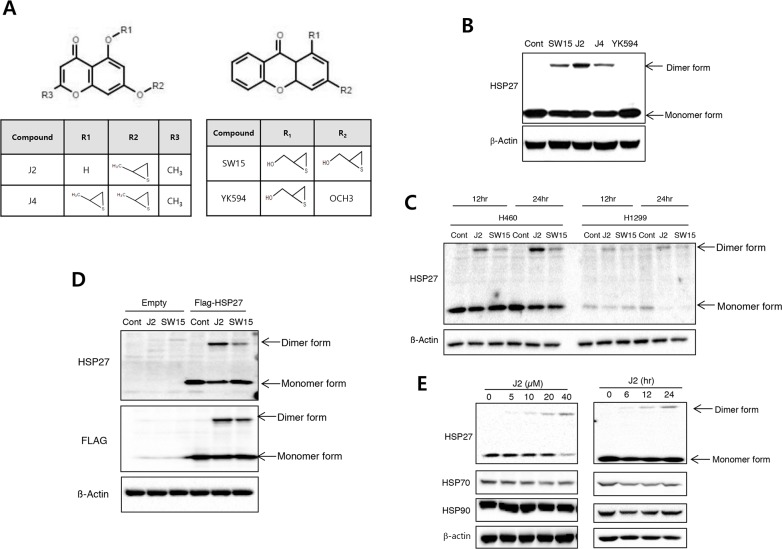
J2 induces more potent HSP27 crosslinking activity than other compounds **(A)** Structures of compounds (J2, J4, SW15 and YK594). **(B)** NCI-H460 cells were treated with SW15, J2, J4 and YK594 at 10 μM for 12 hr and cell lysates were detected by Western blots. **(C)** NCI-H460 or NCI-H1299 cells were treated with J2 or SW15 at 10 μM for 12 or 24 hr and cell lysates were detected by Western blots. **(D)** After transfection with Flag-tagged HSP27, NCI-H460 cells were treated with J2 or SW15 at 10 μM for 12 hr and cell lysates were detected by Western blots. **(E)** NCI-H460 cells were treated with J2 at different concentrations (0, 5, 10, 20 and 40 μM for 12 hr) or times (0, 6, 12 and 24 hr for 10 μM) and cell lysates were detected by Western blot analysis.

Our previous study showed that SW15 promotes cross-linking of HSP27 to form altered dimers, inhibiting oligomerization of HSP27 [13]. For sequential further studies for screening of more potent HSP27 cross linkers using synthetic compounds (Supplementary Figure 1A) in NCI-H460 cells, we selected 3 chromenone compounds with differing side chain structures, J2, J4 and YK-598-2 because they promoted different amounts of altered cross-linking of HSP27 in both cells and HSP27 protein system (Figure 1A and Supplementary Figure 1B and 1C). In the case of YK598-2, cross linking activity was strong, however, cytotoxic activity was also stronger than J2; J2 did not show any strong cytotoxicity at 20 μM. However, YK598-2 showed strong cytotoxicity even at 3 μM. Therefore, we excluded YK-598-2 in this study (Supplementary Figure 1D). When we also compared HSP27 cross linking activity of J2 with SW15 or J4, J2 displayed stronger cross-linking activity than SW15 and J4 (Figure 1B). To elucidate whether cross linking activity of J2 is specific for HSP27, HSP27 high expressed lung cancer cells, NCI-H460 and HSP27 low expressed lung cancer cells, NCI-H1299 cells was compared with HSP27 cross linking activity of J2 and found that J2 showed stronger cross linking activity in HSP27 high expressed lung cancer cells and J2 was more profound activity than SW15 (Figure 1C). When Flag tagged HSP27 was overexpressed to HSP27 low expressed cells, NCI-H1299 cells, HSP27 cross linking activity was dramatically increased (Figure 1D). J2 also showed cross-linking of HSP27 in a time- and dose-dependent manner (Figure 1E). The IC50 value of J2 in NCI-H460 cells was 99.27 ± 1.13 when it was examined by MTT assay, which showed relatively low cytoxicity. In the case of SW15, IC50 was 23.87 ± 1.13, which was more cytotoxic but lower cross linking activity than J2 (Table 1).

**Table 1:** Cytotoxicity of compounds NCI-H460 cell were treated with the different concentrations of compounds for 24 hr and cell viability was analyzed by MTT assay. The table shows the IC50 values (mean ± SD)

Compound	IC50 (μM)
J2	99.27 ± 1.13
SW15	23.87 ± 1.83
YK594	>80
17-AAG	>60
Taxol	0.12 ± 0.02
Cisplatin	>100
HSP9	26.19 ± 1.12
HSP16	>100
RP101	>200

### J2 with more cross linking activity of HSP27 showed more sensitization effects than SW15 in combination with conventional anticancer drugs

**Figure 2:**
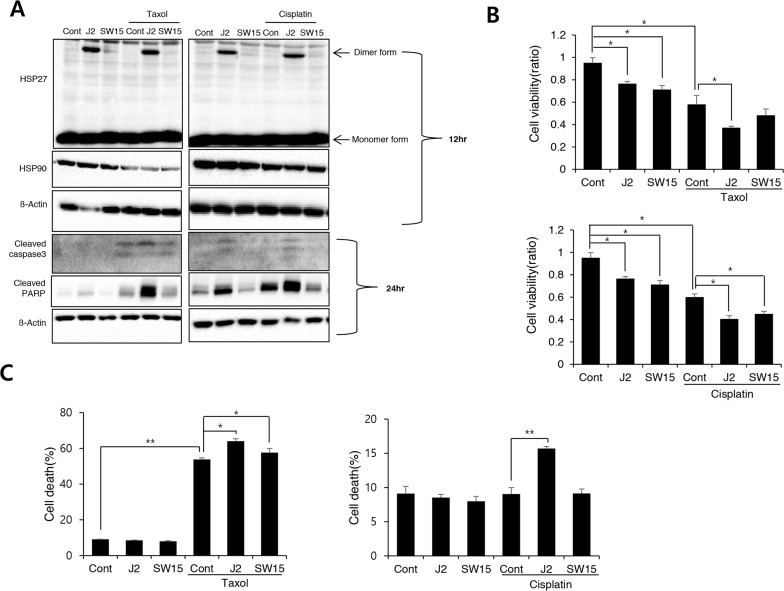
J2 induces more potent sensitization to cancer cells than SW15 **(A)** NCI-H460 cells were treated with J2 (10 μM) and SW15 (10 μM) for 12 and 24 hr, with or without taxol (0.01 μM) or cisplatin (3 μM) and cell lysates were detected by Western blot analysis. Cell viability by MTT assay **(B)** or Annexin V/7-AAD double staining for detection of both apoptosis and necrosis **(C)** was analyzed at 48 hr. Results are the means and standard deviations of three independent experiments (^*^p<0.05 and ^**^p<0.01).

**Figure 3:**
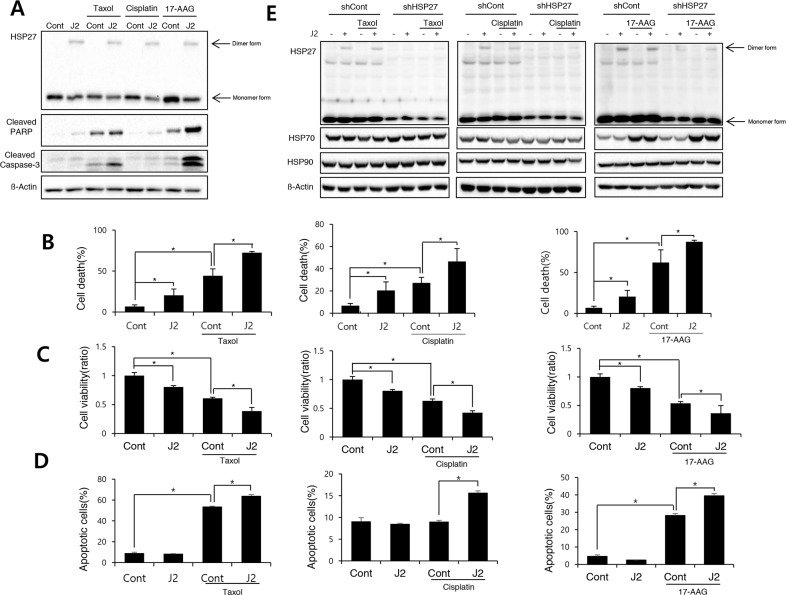
J2 induces sensitization to cancer cells in combination with anticancer drugs **(A)** NCI-H460 cells were treated with J2 (10 μM) for 24 hr, with or without taxol (0.01 μM), cisplatin (3 μM) or 17-AAG (3 μM) and cell lysates were detected by Western blot analysis. **(B)** Cell death was analyzed by flow cytometry after propidium iodide (PI) staining for 48 hr. **(C)** Cell viability was analyzed by MTT assay for 48 hr **(D)** Annexin V staining was performed at 48 hr of the treatments. **(E)** NCI-H460 cells stably transfected with control (sh-Control) and shRNA of HSP27 (sh-HSP27) were treated with or without taxol (0.01 μM), cisplatin (3 μM) or 17-AAG (3 μM) and cell lysates were detected by Western blot analysis. Results are the means and standard deviations of three independent experiments (^*^p<0.05).

Taxol, cisplatin and 17-AAG are potent anticancer drugs, however, resistance emerges early due to compensatory mechanisms involving increased expression of HSP27, which inhibits drug effectiveness [14–17]. To elucidate whether cross linking activity of J2 is correlated with sensitization effects, we compared J2 and SW15 for sensitization effects in combination with taxol, cisplatin or HSP90 inhibitor, 17-AAG. J2, at 10 μM showed more increased cross linking activity of HSP27 than SW15 at same concentration as J2 and when they were combined with taxol, cisplatin, or 17-AAG, sensitization effects were more potently induced in J2 co-treated cells. These effects were shown in detection of cleaved PARP-1, and cleaved caspase-3 and Annexin V/7-AAD double staining for detection of both apoptosis and necrosis, as well as MTT assay data for cell viability (Figure 2). Based on the IC50 values, the doses of taxol, cisplatin and 17-AAG were selected as 0.01 μM, 3 μM and 3 μM, respectively, because these concentrations did not induce more than 30 % cell death after 24 hr of the treatments. Cleaved casepase-3 and PARP cleavage data suggested that 3 hr pretreatment of J2 before treatment of taxol, cisplatin or 17-AAG synergistically sensitized the lung cancer cells at 24 hr of the treatments (Figure 3A). PI staining for total cell death detection, MTT assay data for cell viability and Annexin V staining for detection of apoptosis, also suggested that J2 potentially sensitized the cancer cells in combination with taxol, cisplatin and 17-AAG when they were detected at 48 hr (Figure 3B, 3C and 3D). Moreover, HSP27 cross-linking activity disappeared when shRNA of HSP27 was stably transfected (Figure 3E), suggesting that altered cross- linking of HSP27 by J2 was HSP27-dependent and J2 may give better combination strategy than SW15 for inhibition of HSP27.

### J2 sensitized lung cancer cells xenografted tumors in combination with conventional anticancer drugs

**Figure 4:**
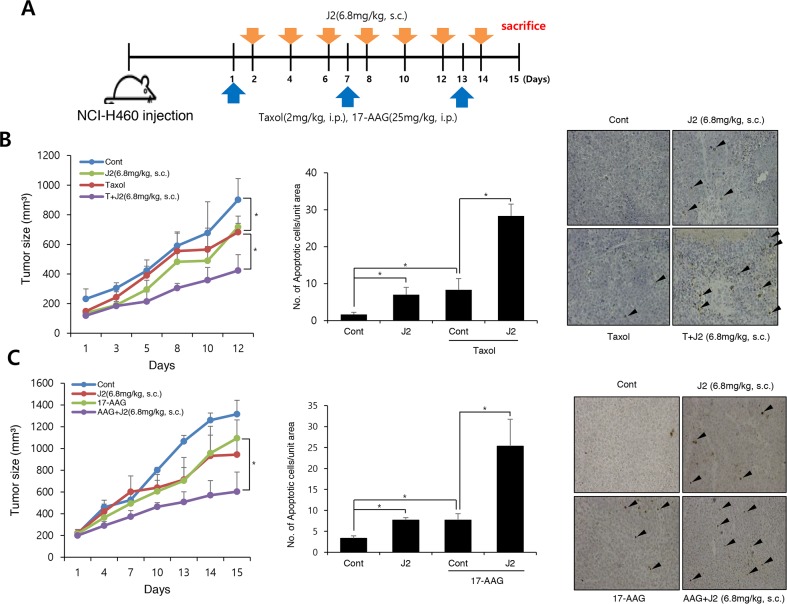
J2 shows synergistic regression effects to xenografted tumors in combination with 17-AAG or taxol **(A)** NCI-H460 cells were injected subcutaneously (s.c.) into BALB/c nude mice (n = 3/group). Xenografted mice were treated 6 times with J2 (6.8 mg/kg) delivered with a local regional application in combined with 2 times intraperitoneal (i.p.) treatment of taxol (2 mg/kg) or 6 times i.p. treatment of 17-AAG (25 mg/kg). **(B, C)** Tumor size was measured twice weekly. Results are the means and standard errors (^*^p<0.05). TUNEL staining were performed using tumor tissues. Arrows indicate TUNEL positive cells. Graph represents mean and standard deviation (^*^p<0.05).

**Figure 5:**
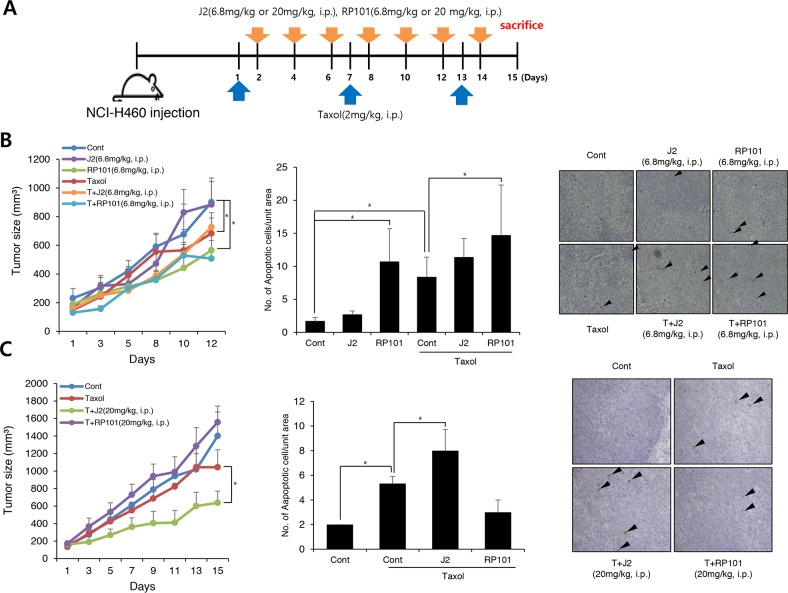
J2 shows more sensitization to xenografted tumor in combination with taxol than RP101 **(A)** NCI-H460 cells were injected subcutaneously (s.c.) into BALB/c nude mice (n = 3/group). Xenografted mice were treated 6 times with J2 (6.8 mg/kg or 20 mg/kg) or RP101 (6.8 mg/kg or 20 mg/kg) delivered with intraperitoneal (i.p.) in combined with 2 times i.p. treatment of taxol (2 mg/kg). **(B, C)** Tumor size was measured twice weekly. Results are the means and standard errors (^*^p<0.05). TUNEL staining were performed using tumor tissues. Arrows indicate TUNEL positive cells. Graph represents mean and standard deviation (^*^p<0.05).

*In vivo* data using nude mice after grafting of NCI-H460 cells indicated that J2 treatment (6.8 mg/kg, subcutaneous local regional application, every other treatment for 3 weeks, s.c.) to tumor site (Figure 4A) led to sensitization in combination with 17-AAG (25 mg/kg, i.p., every other day treatment for 3 weeks) or taxol (2 mg/kg, i.p., once a week for 3 weeks). In this experiment, J2 was s.c. injected to mice for detection of direct J2 effect to tumors with high expression of HSP27. TUNEL-positive areas in tumor tissues also correlated well with the sensitizing effects of J2 in combination with taxol or 17-AAG (Figure 4B and 4C). Secondly, we also elucidated the sensitizing effects of J2 by i.p. administration, because i.p. injection was more favorable rout of drug development. In this experiment, we compared sensitizing effects of J2 with the effects of RP101, a small molecule HSP27 inhibitor which is under the phase II clinical trial [8]. RP101 alone did not show any strong cytotoxicity and IC50 value was >200 μM in NCI-H460 cells (Table 1). i.p. injection of J2 at 6.8 mg/kg which concentration (Figure 5A) was same in that of s.c. injection experiment, did not show any anticancer activity. In the case of RP101 treatment at 6.8 mg/kg, anticancer activity was shown by RP101 alone treatment and taxol combination did not show any increased anticancer activity. Detection of TUNEL positive cells in tumor tissues suggested that 6.8 mg/kg i.p. injection of RP101 alone induced high amount of apoptosis, and taxol combination also significantly induced additional apoptosis even though additional tumor regression effects were not obvious. In the case of i.p. injection of J2 at 6.8 mg/kg, J2 alone did not induce any apoptosis, however, when combined with taxol, apoptosis induction was significantly increased than taxol alone treatment (Figure 5B). When we increased dosage of J2 to 20 mg/kg, synergistic growth regression was shown even by i.p. injection of J2 with synergistically increased TUNEL positive regions. However, in the case of RP101, 20 mg/kg of RP101 treatment alone, differently with 6.8 mg/kg, did not show any anticancer effects and combined sensitization effects with taxol was not also observed (Figure 5C), suggesting different dosage response between J2 and RP101. Significant toxicity was not seen by i.p. injection of J2 or RP101 even at 20 mg/kg high dosage. From the data, J2 with relatively high dose can be used as a sensitizer for injection purpose to abrogate HSP27-mediated resistance.

### 4-Pyron structure of J2 was a pharmacophore for altered cross linking activity of HSP27 by J2

**Figure 6:**
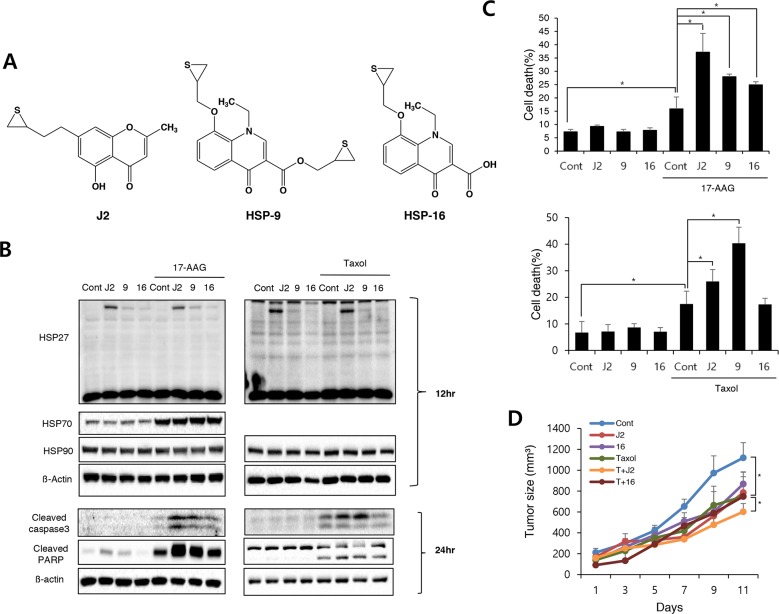
Replacement of chromone with quinolone structure of J2 eliminates cross linking activity **(A)** Structures of the compounds (J2, HSP9, and HSP16). **(B)** NCI-H460 cells were treated with J2 (10 μM), HSP9 (10 μM), HSP16 (10 μM) for 12 and 24 hr, with or without 17-AAG (3 μM) or taxol (0.01 μM) and cell lysates were detected by Western blot analysis. **(C)** Cell death was analyzed by flow cytometry after propidium iodide (PI) staining for 48 hr, with or without 17-AAG (3 uM) or taxol (0.01 μM). Results are the means and standard deviations of three independent experiments (^*^p<0.05). **(D)** Xenografted mice (n = 3/group) were treated 6 times with J2 and HSP16 (20 mg/kg) delivered with intraperitoneal (i.p.) treatment in combined with 2 times i.p. treatment of taxol (2 mg/kg). Tumor size was measured twice weekly. Results are the means and standard errors (^*^p<0.05).

To increase the drug ability of J2 such as pharmacokinetics, solubility and bioavailability, we tried to find alternative efficient HSP27 cross linkers. In this purpose, quinolone, an (bio) isostere of chromone, was designed and synthesized. Because quinolone structures are well-known core in many marketed medicine, especially fluoroquinolone antibiotics, we expected that replacement of oxygen with nitrogen improve the drug ability issue of J2 (Supplementary Figure 2). Thioepoxide ring, however, was kept since it is necessary for the inhibitory activity against altered HSP27 cross linking activity. We have synthesized quinolone derivatives such as HSP9 or HSP16 (Figure 6A) and evaluated altered HSP27 cross linking activities. However, these quinolone compounds did not show any altered HSP27 cross linking activity at all (Figure 6B). Moreover, sensitizing activity of HSP9 or HSP16 was examined to evaluate whether altered HSP27 cross linking activity was correlated with sensitizing effects. As expected, HPS9 and HSP16 showed less sensitizing effects than J2 when they were combined with 17-AAG, however, in the case of HSP9, more sensitization activity than J2 was observed when it was combined with taxol, suggesting HSP27 independent cytotoxic activity by HSP9 (Figure 6B and 6C). Indeed IC50 of HSP9 was much lower than that of J2 (Table 1). Because of HSP27 independent effects and relative high cytotoxicity, we excluded HSP9 for *in vivo* study. More tumor regression by J2 combination with taxol was observed *in vivo* tumor cells xenograft model, while HSP16 was not (Figure 6D), suggesting quinolone structure may be a pharmacophore for induction of altered HSP27 cross linking, because in the case of pyridine which is the replacement oxygene radical of pyrone residue for chromone to nitrogen, altered HSP27 cross linking activity was disappeared.

## DISCUSSION

HSP27 is a stress-induced protein which acts as a molecular chaperone and plays a role in the inhibition of apoptosis. The negative effects of HSP27 is the result of its ability to modulate key steps of the apoptotic cascade through interaction with crucial regulators such as cytochrome c [18], pro-caspase-3 [18], DAXX [19], Akt [18], Stat3 [20], eIF4E [18, 21], PKCdelta [22, 23] and F-actin [18], an upstream modulator of apoptosis. In many solid tumors, HSP27 levels correlate with poor prognosis. Unlike other HSPs such as HSP70 or HPS90, HSP27 haven't ATP-binding site, making HSP27 a difficult target. Hence, although HSP27′s importance as cancer target, currently only two cancer therapy approaches such as OGX427, antisense of HSP27 and RP101, a small molecule, targeting HSP27 are under clinical investigation. In this study, we suggested a small molecule, J2, the following ZER and SW15 that forms altered cross-linking of HSP27 without any serious cellular toxicity, which induces more strongly formation of altered dimerization of HSP27. Considering that the only difference between J2 and J4 is R1 residue, however, dimerized effect was stronger in J2 than J4. So, we selected J2 as a candidate for HSP27 inhibitor, suggesting differences in the detailed chemical structure may cause differences in HSP27 dimerization activity.

Altered dimerization activity was stronger in J2 than SW15, however, cellular cytotoxicity was weaker in J2 than SW15, suggesting that J2 is expected to be more useful when combined with cytotoxic anticancer drugs including taxol, cisplatin and 17-AAG for cancer cells overexpressing HSP27. The combination treatment of J2 with taxol, cisplatin and 17-AAG induced great sensitization, suggesting that J2 might have clinical implications as an adjuvant universal HSP27 inhibitor as a sensitizer to combat HSP27-mediated resistance. Moreover, cross-linking of HSP27 by J2 was specific for HSP27 protein, because cross-linking activity in sh-HSP27 cells disappeared. The treatment of J2 alone did not show any strong cytotoxicity *in vitro* assay, however, *in vivo* data revealed that anti-tumor activity of J2 is similar to the effects of taxol or 17-AAG, which may give a stronger rationale to J2 as a candidate of anticancer drug, especially in combination therapy.

More interesting points for J2 as an anticancer drug for abrogation of HSP27 may be the possibility of i.p. administration. High dose of J2, 20 mg/kg even at i.p. administration, induced sensitization in xenograft tumor model, while low dose of J2, 6.8 mg/kg did not even though TUNEL positive cells of 6.8 mg/kg J2 treated tumors were increased. Of course, even though more detailed pharmacokinetics approaches should be needed, these data suggested J2 as a possible candidate drug for sensitization as combination with conventional anticancer drugs in HSP27 overexpressed lung cancer patients.

To promote better druggability, we tried to find alternative efficient derivatives of J2. In this consideration, quinolone compounds, such as HSP9 and HSP16 have been synthesized and evaluated their biological activities. Unexpectedly, HSP9 and HSP16 with reduced cross linking activity of HSP27, showed less sensitization in combination with 17-AAG than J2. However, when taxol was combined, HSP9 but not HSP16 showed more sensitization than J2 even though crosslinking activity was less than J2. One possibility of this effect may be HSP27 independent cytotoxicity by HSP9. Indeed, IC50 value of HSP9 was much lower than J2. Another possibility is that because cellular resistance by 17-AAG more related to HSP27 overexpression than taxol, abrogation of HSP27 can more effectively sensitize the cancer cells.

In conclusion, since over-expression of HSP27 induces cancer cells resistant to many cancer treatments and HSP27 expression correlates with poor survival of lung tumor patients, especially those with lung adenocarcinoma, inhibition of HSP27 is a good strategy for sensitization of NSCLC in combination with other anticancer drugs. Small molecule, J2, which promotes altered cross-linking of HSP27, offer a promising approach for inhibition of HSP27, although further research is needed to evaluate the safety and efficacy of this compound in a clinical setting. However, one thing that J2 is more advanced than the previous ones such as SW15 or zerumbone [12, 13] is that it can be used for i.p. injection in combined with conventional anticancer drugs.

## MATERIALS AND METHODS

### Compounds and chemicals

Compounds were synthesized as described previously [13, 24] or as in Supplementary Information. Taxol (Santa Cruz, sc-201439A), and tanespimycin (17-N-allylamino-17-demethoxygeldanamycin, 17-AAG) (Selleckchem, S1141) were dissolved in DMSO and diluted in cell culture medium. Cisplatin (Sigma Aldrich, P4394) was dissolved in PBS and diluted in cell culture medium.

### Cell culture and transfection

Human non-small cell lung cancer cell line NCI-H460 was cultured in RPMI supplemented with 10% fetal bovine serum and 1% penicillin streptomycin in a 37°C incubator with 5% CO_2_. Transfections were performed using Lipofectamine 2000 (Invitrogen). Lentiviruses were used to create stable NCI-H460 cell lines expressing shRNA for HSP27 with a puromycin-resistance gene. The HSP27 shRNA plasmid (sc-2935-SH) and shRNA Plasmid Transfection Reagent (sc-108061) were from Santa Cruz Biotechnology. To generate the sh-control and sh-HSP27 cells, cell lines were transduced with 1 mol lentivirus and selected using puromycin (1 μg/mL) for at least one week.

### Antibodies

Goat polyclonal anti-HSP27 (sc-1049), goat polyclonal anti-HSP70 (sc-1060), mouse monoclonal anti-β-actin (sc-47778), and mouse monoclonal anti-HSP90 (sc-13119) were from Santa Cruz Biotechnology. Rabbit polyclonal anti-cleaved caspase-3 (#9661), and rabbit polyclonal anti-cleaved PARP (#9541) were from Cell Signaling. Rabbit monoclonal anti-HSF1 (ab52757) antibodies were from Abcam.

### Polyacrylamide gel electrophoresis and western blots

For polyacrylamide gel electrophoresis (PAGE) and Western blots, cells were lysed with radioimmunoprecipitation assay buffer (50 mM Tris-HCl pH 7.5, 150 mM NaCl, 1% NP-40, 0.1% sodium dodecyl sulfate [SDS], and 1% sodium deoxycholate) supplemented with 1 mM Na_3_VO_4_, 1 mM dithiothreitol, 1 mM phenylmethylsulfonyl fluoride, and protease inhibitor cocktail (Calbiochem). Samples were boiled for 5 min, and an equal amount of protein was analyzed on SDS-PAGE.

### Annexin V/7-AAD staining analysis

The cells were collected by trypsin-EDTA and centrifuged at 1300 rpm 3min. Following resuspension in binding buffer (10 mM HEPES-NaOH, 140 mM NaCl, 2.5 mM CaCl_2_) 100 ul of a single-cell suspension was incubated with 5 μl Annexin V-PE and 5 μl 7-AAD for 15 min at room temperature in the dark. After addition of 400 μl of binding buffer, the samples were analyzed with a BD FACS Calibur flow cytometer (BD Bioscience, San Jose, CA, USA) within 1 hr. For each sample, 10000 events were counted.

### MTT analysis

The 3-(4,5-dimethyl-2-thiazolyl)-2,5-diphenyl-2H-tetrazolium bromide, methylthiazolyldiphenyl-tetrazolium bromide (MTT) (Sigma Aldrich, M5655) assay was used as an indirect measure of cell viability. For MTT assays, cells were seeded in 96-well plates overnight and treated with drugs for 24 hr at 37°C with 5% CO_2_. After incubation, cell viability was determined by adding 100 μl MTT solution (5 mg/ml in PBS) to wells followed by incubation for 4 hr at 37°C with 5% CO_2_. MTT mixtures were removed and 100 μl DMSO was added to wells. Samples were shaken for 30 min, and absorbance at 540 nm was recorded for ELISAs. Cell viability was calculated as follows: 1-average absorbance of treated group/average absorbance of control group.

### Flow cytometry analysis

Cells were washed twice with 1× phosphate buffered saline (PBS) and dissociated using trypsin-EDTA and centrifuged at 13,000 rpm for 3 min at 4°C. After adding 1 ml 1x PBS and 10 μg/ml propidium iodide to samples in polystyrene round-bottom tubes, flow cytometric analysis was performed using a BD FACS Calibur flow cytometer (BD Bioscience, San Jose, CA, USA).

### Tumor xenografts in nude mice

Single-cell suspensions (1 × 10^6^ cells) were injected subcutaneously into hind legs of 6-week-old BALB/c athymic nude mice (SLC, Hamamatsu, Japan). When tumors reached a minimal volume of 100 mm^3^, xenografted mice were treated 6 or 9 times with J2 by local regional application with or without treatment with 17-AAG (25 mg/kg) or taxol (2 mg/kg or 3 mg/kg) by i.p. injection. Injection of i.p. of J2 or RP101 (6.8 mg/kg or 20 mg/kg) was performed once every 2 days. Tumor volumes were determined according to the formula (L x l^2^)/2, by measuring tumor length (L) and width (l) with a caliper. Tumors were measured every other day and allowed to grow for 3 weeks.

### TUNEL assays

For TUNEL assays, Peroxidase *In Situ* Apoptosis Detection kits (Millipore, 17-141) were used according to the manufacturer's recommendations. The number of TUNEL-positive tumor cells and the total number of tumor cells were measured for three microscopic fields of randomly selected tumors and mean value calculated as percentage of TUNEL-positive tumor cells.

### Statistical analysis

Values are displayed as mean plus or minus standard deviation or standard error. Comparisons between groups were by one-way ANOVA for experiments with more than three subgroups. Post hoc range tests were performed with one-way ANOVA. Results were considered statistically significant for *p* values less than 0.05.

## SUPPLEMENTARY MATERIALS FIGURES


