# Supplementary material for: Sensitization of lung cancer cells by altered dimerization of HSP27
Source: Oncotarget. 2017 Oct 31;8(62):105372–82. doi: 10.18632/oncotarget.22192 (PMC5739644; doi:10.18632/oncotarget.22192)
Supplement: Supplementary file 1 [file oncotarget-08-105372-s001.pdf]

Sensitization of lung cancer cells by altered dimerization of HSP27

SUPPLEMENTARY MATERIALS

General synthesis

The solvents and reagents used were of the highest commercial grade available and used as received. The TLC plates were Kieselgel 60 F254 (art A715, Merck). Silica gel 60 (0.040−0.063 mm ASTM, Merck) was used for column chromatography. The ^{1}H- and ^{13}C-NMR spectra were recorded on a Varian NMR AS 400 MHz instrument. Chemical shifts (δ) are in parts per million (ppm) relative to tetramethylsilane as an internal standard, and coupling constants (*J* values) are in Hertz. Mass spectral investigations were performed on an Agilent 6430 equipped with an electrospray ionization (ESI) source. The melting points were measured on Gallenkamp Melting Point Apparatus without correction.

Synthesis of compounds

Dimethyl 2-(((2-methoxyphenyl)amino)methylene) malonate (1)

A reaction mixture of *o*-anisidine (2.00 g, 16.23 mmol) and dimethyl methoxymethylene malonate (2.83 g, 16.23 mmol) in methanol (10 mL) was stirred at RT for 2 h and then solvent was reduced under reduced pressure. Solid formed was filtered and washed with methanol and then dried under vacuum to give compound **1** (3.26 g, 75.1%) as a white solid. R_f 0.75 (ethyl acetate : *n*-hexane = 1 : 1); ^{1}H-NMR ($CDCl_3$, 400 MHz) δ 3.78 (s, 3H), 3.87 (s, 3H), 3.95 (s, 3H), 6.94 (dd, *J* = 8.0, 1.2 Hz, 1H), 6.99 (ddd, *J* = 8.0, 8.0, 0.8 Hz, 1H), 7.11 (ddd, *J* = 8.0, 8.0, 0.8 Hz, 1H), 7.26 (dd, *J* = 8.0, 1.6 Hz, 1H), 8.58 (d, *J* = 13.6 Hz, 1H), 11.23 (d, *J* = 13.6 Hz, 1H); ^{13}C-NMR ($CDCl_3$, 100 MHz) 51.7, 51.8, 56.2, 93.3, 111.5, 114.7, 121.4, 125.3, 128.8, 149.2, 151.1, 166.4, 169.3 ppm.

Dimethyl 2-((ethyl(2-methoxyphenyl)amino) methylene)malonate (2)

To a reaction of compound **1** (3.26, 12.29 mmol) and NaOH (0.74 g, 18.44 mmol) in DMSO (20 mL) was added iodoethane (5.75 mL, 36.87 mmol). The reaction mixture was stirred at RT for 4 h and then water was added. After extraction with ethyl acetate, organic solvent was washed with water, and then dried over anhydrous $MgSO_4$. Solvent was removed under reduced pressure and residue

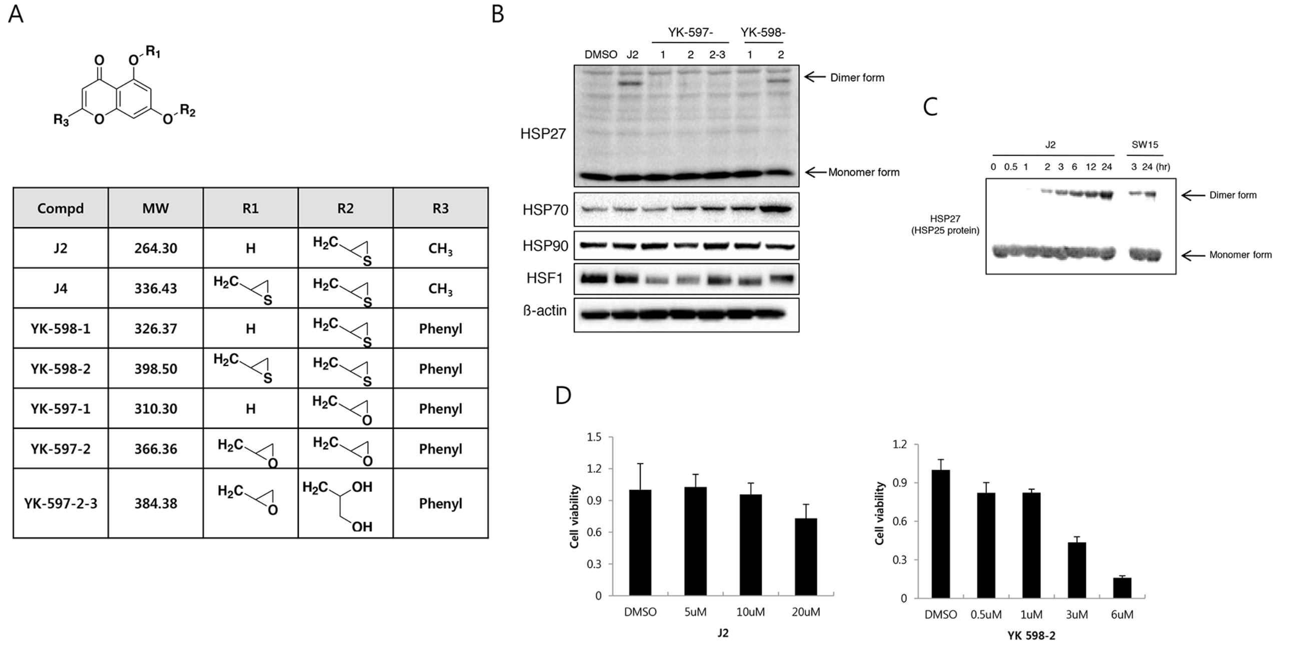

Supplementary Figure 1: (A) Structures of compounds **(B)** NCI-H460 cells were treated with YK597s and YK598 at 10 µM for 12 hr and cell lysates detected by Western blots. **(C)** Murine recombinant wild type HSP25 protein after treatment with the 0.5 mM J2 or 0.5 mM SW15 at indicated time points was analyzed by SDS-PAGE. **(D)** Cell death after 24 hr treatment was analyzed by flow cytometry after propidium iodide (PI) staining.

was purified by silica gel column chromatography (eluant: Ethyl acetate : *n*-Hexane = 1 : 2) to give compound **2** (2.01 g, 55.8%) as a colorless liquid. R_f 0.43 (Ethyl acetate : *n*-hexane = 1 : 1); ^{1}H-NMR ($CDCl_3$, 400 MHz) δ 1.15 (t, J = 7.2 Hz, 3H), 3.01 (s, 3H), 3.60-3.63 (m, 2H), 3.68 (s, 3H), 3.86 (s, 3H), 6.91-6.96 (m, 2H), 7.08 (dd, J = 8.0, 1.6 Hz, 1H), 7.24-7.29 (m, 1H), 7.73 (s, 1H).

Methyl 1-ethyl-8-methoxy-4-oxo-1,4-dihydroquinoline-3-carboxylate (3)

The reaction mixture of compound **2** in Eaton reagent(15 mL) was stirred at 80 °C for 5 h. After completion of reaction, the reaction mixture was cooled to RT and pH was adjusted to 5-6 with sat-Na_2CO_3. After extraction with ethyl acetate, organic solvent was washed with water, and then dried over anhydrous $MgSO_4$. Solvent was removed under reduced pressure and residue was purified by silica gel column chromatography (eluent:MeOH:$CHCl_3$ = 1:20) to give compound **3** (1.41 g, 78.8%) as an ivory solid. R_f 0.47 (MeOH:$CHCl_3$ = 1:9); ^{1}H-NMR (DMSO-d_6, 400 MHz) δ 1.32 (t, J = 6.8 Hz, 3H), 3.75 (s, 3H), 3.97 (s, 3H), 4.59 (q, J = 6.8 Hz, 2H), 7.41-7.42(m, 2H), 7.88 (dd, J = 5.6, 3.6 Hz, 1H), 8.55 (s, 1H); ^{13}C-NMR ($CDCl_3$, 100 MHz) 16.9, 52.3, 54.8, 56.6, 110.5, 114.8, 120.3, 121.2, 125.7, 130.1, 132.2, 150.0, 151.5 ppm.

1-Ethyl-8-hydroxy-4-oxo-1,4-dihydroquinoline-3-carboxylic acid (4)

To a reaction mixture of compound **3** (0.5 g, 1.91 mmol) in CH_2Cl_2 (20 mL) was added BBr_3 (0.92 mL, 9.55 mmol). The reaction mixture was stirred at RT for 12 h. The pH of reaction mixture was adjusted to 5-6 with sat-$NaHCO_3$and then extracted with dichloromethane-ethyl acetate. Organic solvent was collected, washed with $H_2O$and then dried over $MgSO_4$. Solvent was removed

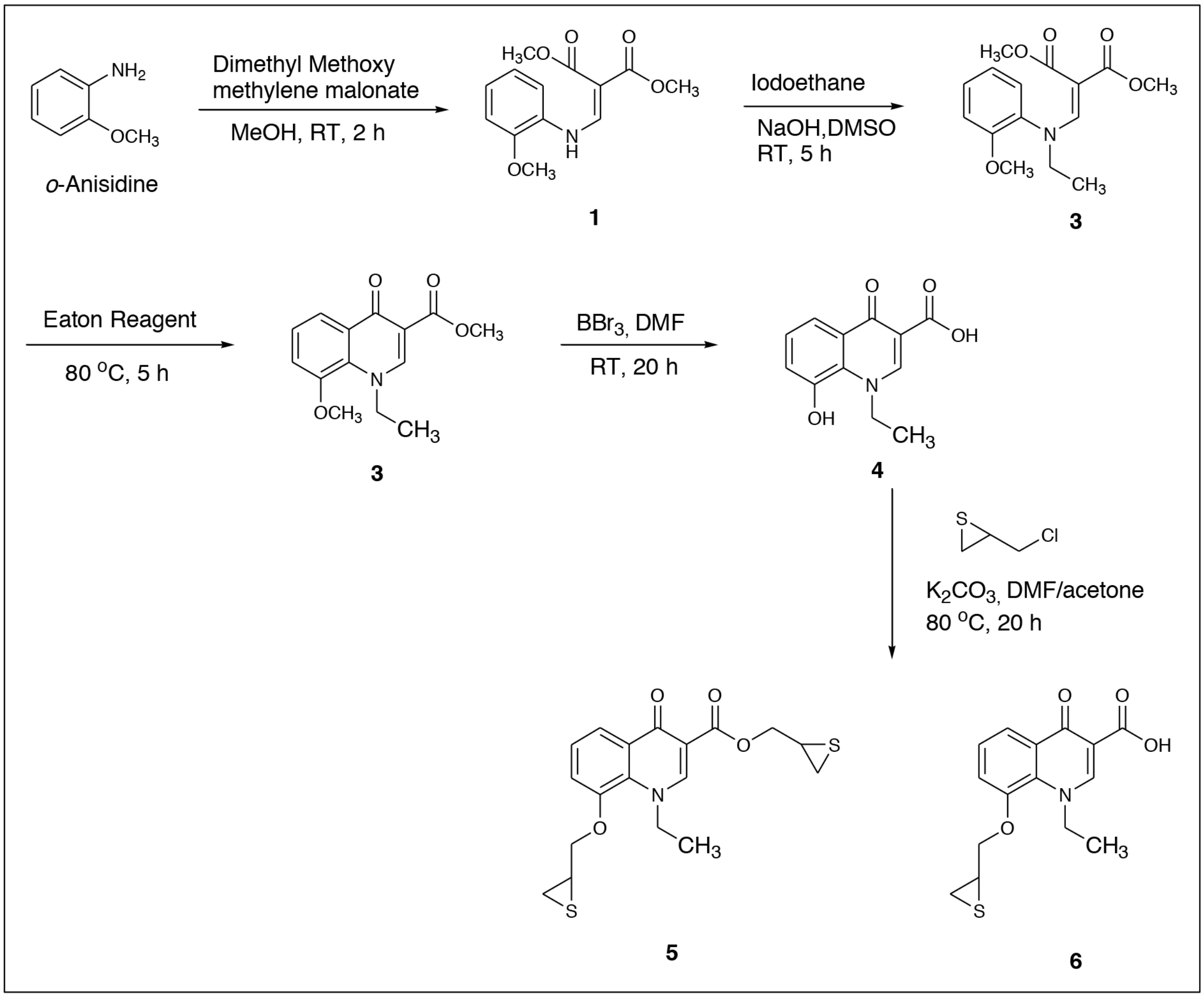

Supplementary Figure 2: Synthesis of compounds.

under reduced pressure and residue was purified by silica gel column chromatography (eluent: MeOH:$CHCl_3$ = 1:30) to give compound **4** (0.10 g, 22.5%) as an ivory solid. R_f 0.15 (MeOH:$CHCl_3$ = 1:9); ^{1}H-NMR (DMSO-d_6, 400 MHz) δ 1.41 (t, *J* = 7.2Hz, 3H), 4.86 (dd, *J* = 7.2 Hz, 2H), 7.36 (dd, *J* = 8.0, 1.6 Hz, 1H), 7.47(t, *J* = 7.6 Hz, 1H), 7.87 (dd, *J* = 8.0, 1.6 Hz, 1H), 8.86 (s, 1H), 11.16 (s, 1H), 15.37 (s, 1H); ^{13}C-NMR (DMSO-d_6, 100 MHz) 17.1, 54.2, 106.9, 116.2, 120.3, 127.1, 128.2, 128.9, 148.8, 150.4, 166.1, 177.4 ppm.

Thiiran-2-ylmethyl 1-ethyl-4-oxo-8-(thiiran-2-ylmethoxy)-1,4-dihydroquinoline-3-carboxylate (5, HSP9)

To a reaction mixture of compound **4** (0.1 g, 0.43 mmol) and K_2CO_3(0.18 g, 0.86 mmol) in acetone (5 mL)/DMF (5 mL) was added epithiochlorohydrin (0.13 mL, 0.65 mmol). The reaction mixture was stirred at 70-75 °C for 16 h and then cooled to RT. Water was added and the mixture was extracted with ethyl acetate and then organic layer was washed with water. Organic solvent was dried over $MgSO_4$ and removed under reduced pressure and residue was purified by silica gel column chromatography (eluent: MeOH:CH_2Cl_2 = 1:30) to give compound **5** (37.7 mg, 25.1%) as a pale yellow semisolid. R_f 0.34 (ethyl acetate:*n*-hexane = 1:1); ^{1}H-NMR ($CDCl_3$, 400 MHz) δ 1.54 (t, *J* = 7.2 Hz, 3H), 2.38 (dd, *J* = 5.2, 1.6 Hz, 1H), 2.45 (dd, *J* = 5.2, 1.2 Hz, 1H), 2.59 (d, *J* = 6.0 Hz, 1H), 2.68 (dd, *J* = 6.0, 1.6 Hz, 1H), 3.29-3.38 (m, 2H), 4.11 (dd, *J* = 10.0, 6.8 Hz, 1H), 4.27 (dd, *J* = 11.6, 7.4 Hz, 1H), 4.36 (dd, *J* = 10.0, 6.0 Hz, 1H) 4.58 (dd, *J* = 12.0, 4.8 Hz, 1H), 4.64 (q, *J* = 7.2 Hz, 2H), 7.12 (dd, *J* = 8.4,1.6 Hz, 1H), 7.34 (dd, *J* = 8.0, 8.0 Hz, 1H), 8.20 (d, *J* = 8.0, 1.6 Hz, 1H), 8.42 (s, 1H); ^{13}C-NMR ($CDCl_3$, 100 MHz) 17.2, 23.4, 24.6, 31.2, 51.1, 54.9, 68.6, 74.4, 110.3, 115.4, 120.8, 125.7, 129.9, 132.2, 148.7, 151.6, 165.7, 173.9 ppm; LRMS-ESI (m/z) $[M+H]^+C_{18}H_{19}NO_4S_2$ calcd 378.08, found 378.20.

1-Ethyl-4-oxo-8-(thiiran-2-ylmethoxy)-1,4-dihydroquinoline-3-carboxylic acid (6, HSP16)

To a reaction mixture of compound **4** (0.1 g, 0.43 mmol) and K_2CO_3 (0.18 g, 0.86 mmol) in acetone (5 mL)/DMF (5 mL) was added epithiochlorohydrin (0.13 mL, 0.65 mmol). The reaction mixture was stirred at 55-60 °C for 16 h and then cooled to RT. Water was added and pH was adjusted to 6-7 and the mixture was extracted with ethyl acetate/CH_2Cl_2. Organic layer was washed with water and then dried over $MgSO_4$. Solvent was removed under reduced pressure and residue was purified by silica gel column chromatography (eluent: MeOH:$CHCl_3$ = 1:40) to give compound **6** (20.5 mg, 15.6%) as a white solid. R_f 0.53 (MeOH:CH_2Cl_2 = 1:20); m.p.; 149-150 °C; ^{1}H-NMR ($CDCl_3$, 400 MHz) δ 1.59(t, *J* = 7.2 Hz, 3H), 2.39 (dd, *J* = 5.2, 1.6 Hz, 1H), 2.70 (dd, *J* = 6.0, 1.6 Hz, 1H), 3.34-3.40 (m, 1H), 4.13 (dd, *J* = 9.6, 7.0 Hz, 1H), 4.43 (dd, *J* = 9.6, 5.6 Hz, 1H), 4.78 (q, *J* = 7.2 Hz, 2H), 7.24 (dd, *J* = 8.0, 1.6 Hz, 1H), 7.49 (dd, *J* = 8.0, 8.0 Hz, 1H), 8.21 (dd, *J* = 8.0, 1.6 Hz, 1H), 8.67 (s, 1H), 14.95 (s, 1H); ^{13}C-NMR ($CDCl_3$, 100 MHz) 17.4, 23.4, 30.9, 55.9, 74.6, 109.0, 116.2, 119.9, 126.8, 129.5, 149.1, 150.6, 167.3, 178.1 ppm; LRMS-ESI (m/z) $[M+H]^+C_{15}H_{16}NO_4S$calcd 306.08, found 306.60.
